# Supplementary material for: Fluorescent Quinolinium Derivative as Novel Mitochondria Probe and Function Modulator by Targeting Mitochondrial RNA
Source: Molecules. 2023 Mar 16;28(6):2690. doi: 10.3390/molecules28062690 (PMC10053327; doi:10.3390/molecules28062690)
Supplement: Supplementary file 1 [file molecules-28-02690-s001.zip › molecules-2258091-supplementary.pdf]

Supplementary Information

Fluorescent Quinolinium Derivative as Novel Mitochondria Probe and Function Modulator by Targeting Mitochondrial RNA

Bo-Zheng Wang †, Ying-Chen Zhou †, Yu-Wei Lin †, Xiu-Cai Chen, Ze-Yi Yu, Yao-Hao Xu, Jia-Heng Tan, Zhi-Shu Huang and Shuo-Bin Chen *

Guangdong Provincial Key Laboratory of New Drug Design and Evaluation, School of Pharmaceutical Sciences, Sun Yat-sen University, Guangzhou 510006, China
* Correspondence: chenshb8@mail.sysu.edu.cn
† These authors contributed equally to this work.

Table of Contents

**Figure S1**	mtDNA copy numbers of RKO cells treated by **QUCO-1**.	S2
**Figure S2**	Systemically evaluation of glycolysis function with **QUCO-1** treatment after 24 h.	S2
**Figure S3**	Co-localization of QUCO-1 and mitochondria in NCM460 with different time and concentration.	S3
**Table S1**	Summarize optical properties of the fluorescent probe	S3

Method of mesuring optical properties of the fluorescent probe S3

^{1}H NMR, ^{13}C NMR and HRMS Spectrum of **QUCO-1** S4

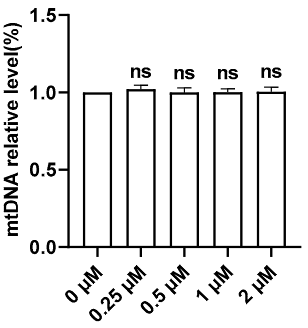

Figure S1. mtDNA copy numbers of RKO cells treated by **QUCO-1**. ns, no significance.

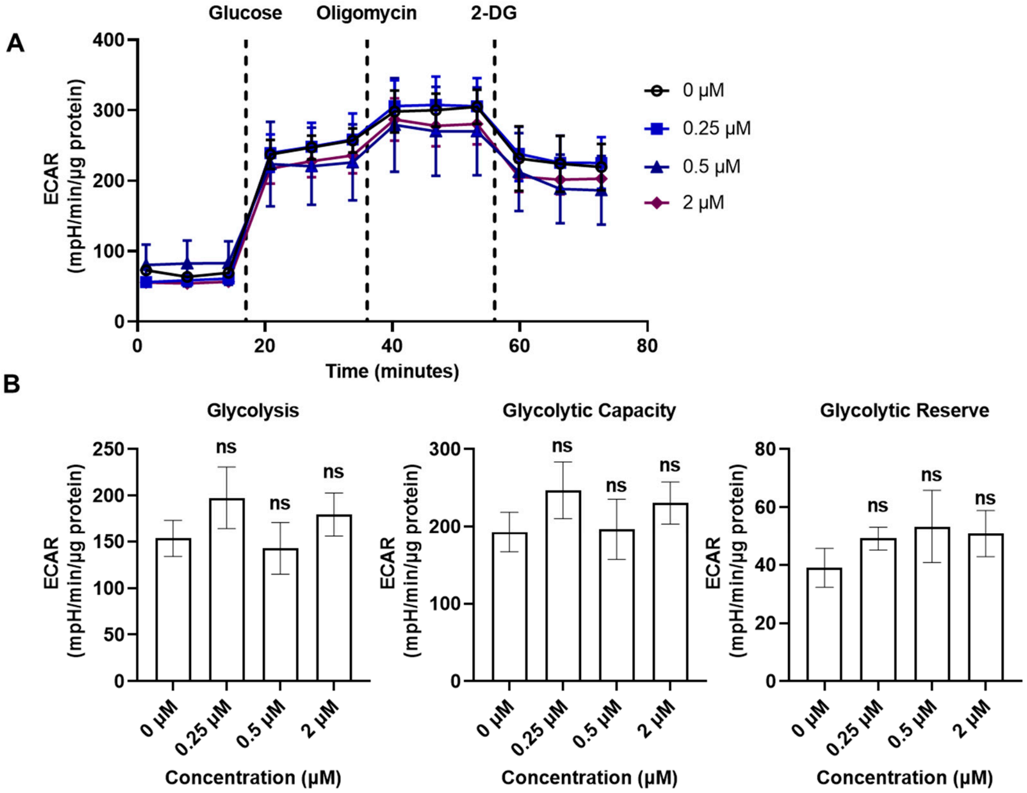

Figure S2. Systemically evaluation of glycolysis function with **QUCO-1** treatment after 24 h. A, extracellular flux was determined in RKO cells cultured in 2 mM glutamine. Representative graphs demonstrate the effects of glucose, olytomycin and 2-DG on EACR. B, the glycolysis, glycolytic capacity and glycolytic reserve calculated by Seahorse XF Cell Mito Stress Test Reporter Generator. Datas are represented as mean ± SEM, n=4, ns, no significance.

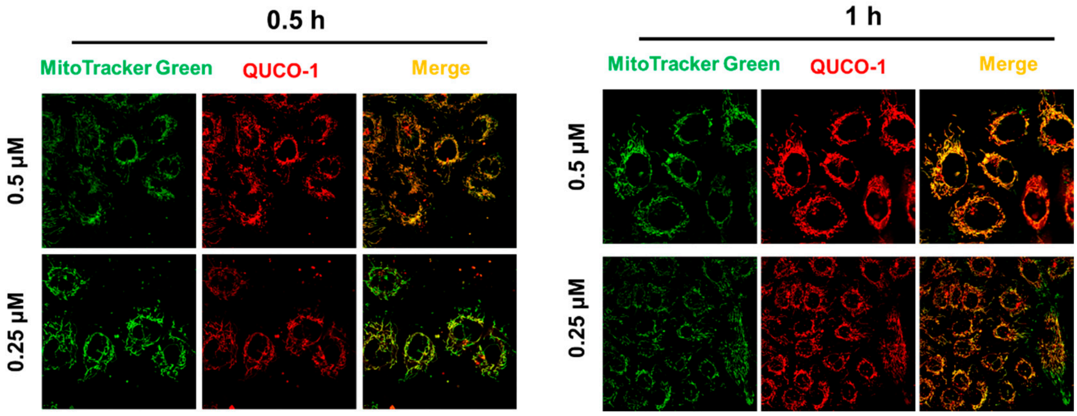

Figure S3. Co-localization of **QUCO-1** and mitochondria in NCM460 with different time and concentration.

Table S1. Summarize optical properties of the fluorescent probe

Solvents	H_2O	MeCN	MeOH	EtOH	EA	DCM
Emission	518	525	523	535	543	556
Absorbance	658	654	651	653	654	639
Stokes' shift	140	129	128	118	111	83
Molar absorptivity	30000	39000	50000	47000	10000	36000
Fluorescence quantum yield	0.006	0.106	0.083	0.126	0.103	0.148

Method of measuring optical properties of the fluorescent probe

Fluorescence studies were performed on Fluoromax-4 Spectrofluorometer (HORIBA, Japan). A quartz cuvette with 2 mm × 10 mm path length was used for the spectra recorded at 1 nm excitation and emission slit widths unless otherwise specified. All parameters were measured and calculated in classic methods. The absolute fluorescence quantum yield (Φ_F) of QUCO-1 was measured by Quanta-Phi module for Fluoromax-4 Spectrofluorometer.

Some ^{1}H NMR, ^{13}C NMR, HRMS and HPLC spectrum of 2, 3, 4 and QUCO-1

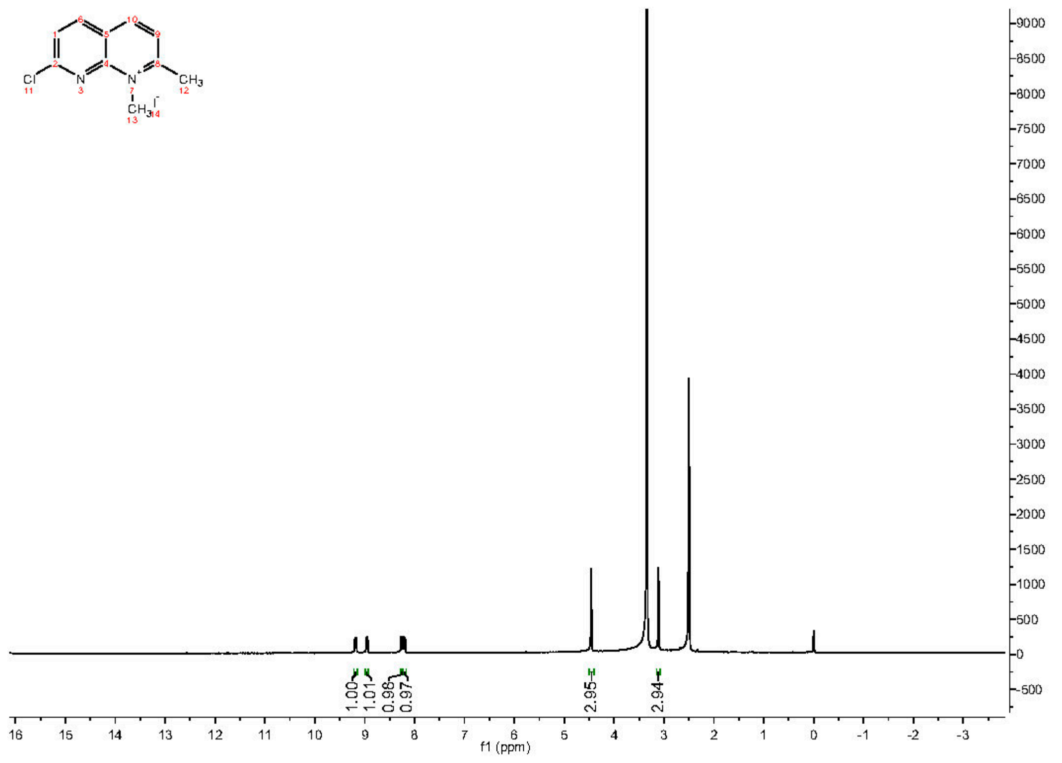

^{1}H NMR spectrum of **2**

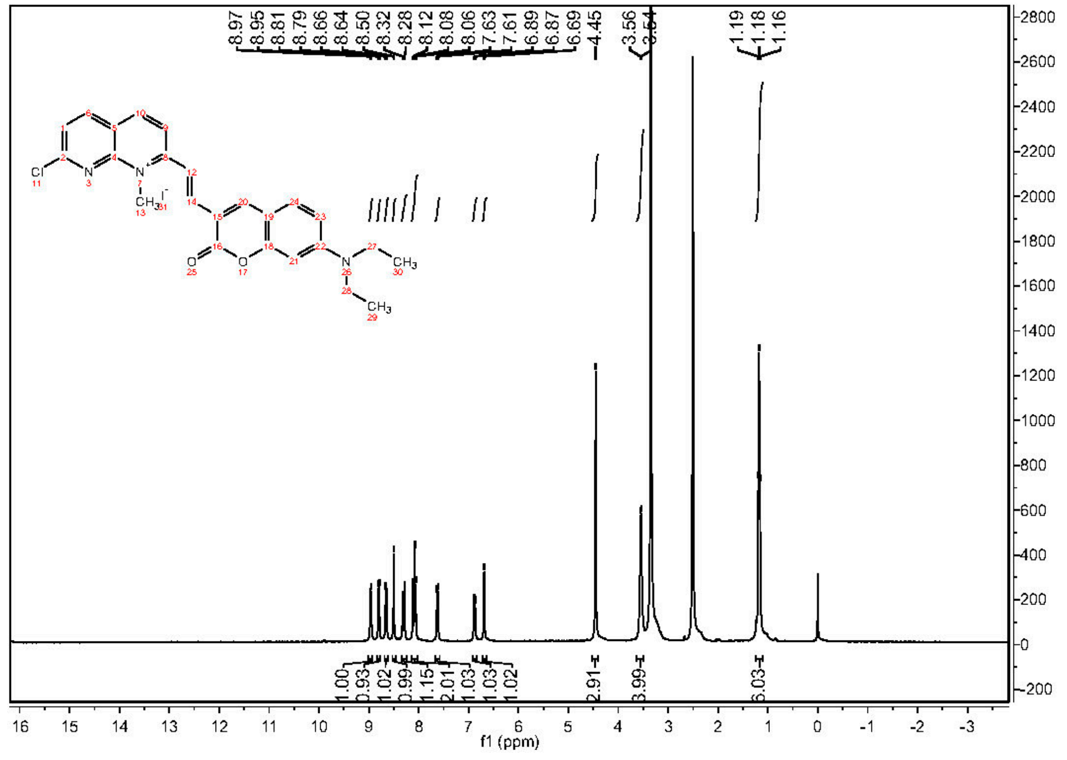

^{1}H NMR spectrum of **3**

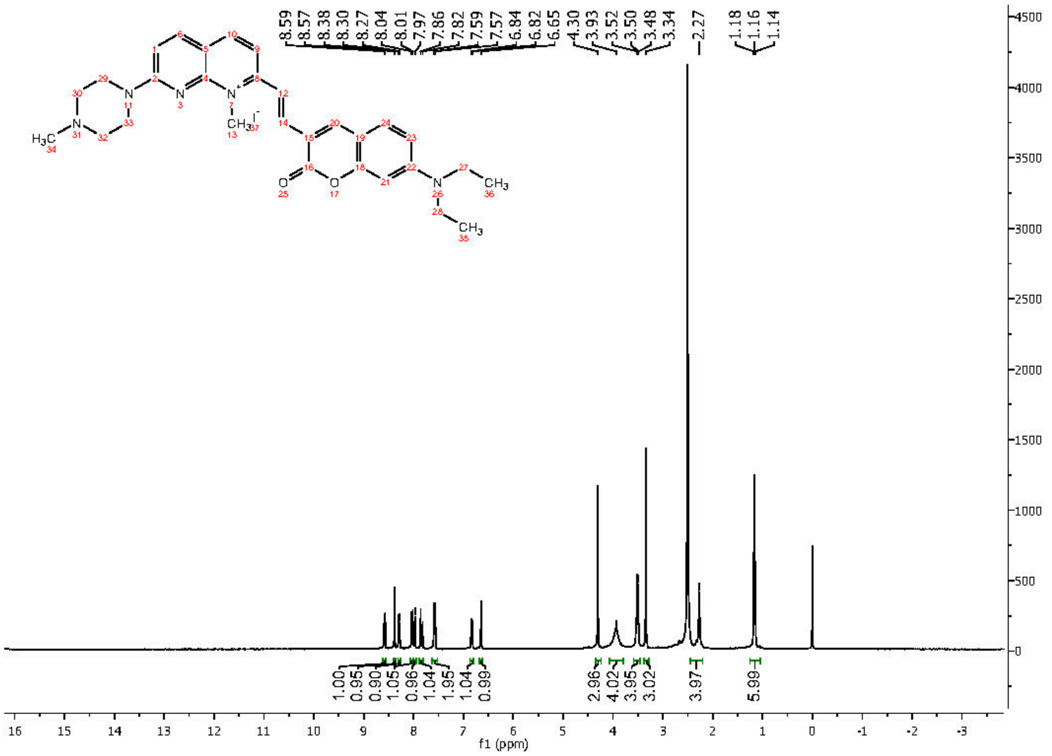

^{1}H NMR spectrum of **QUCO-1**

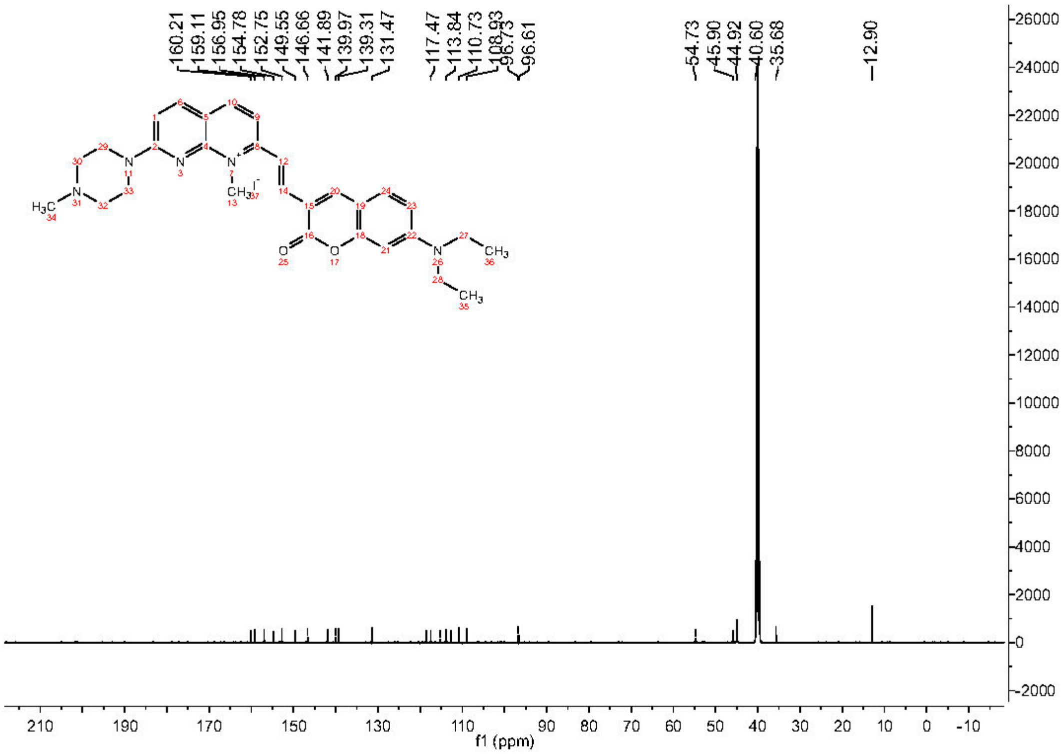

^{13}C NMR spectrum of **QUCO-1**

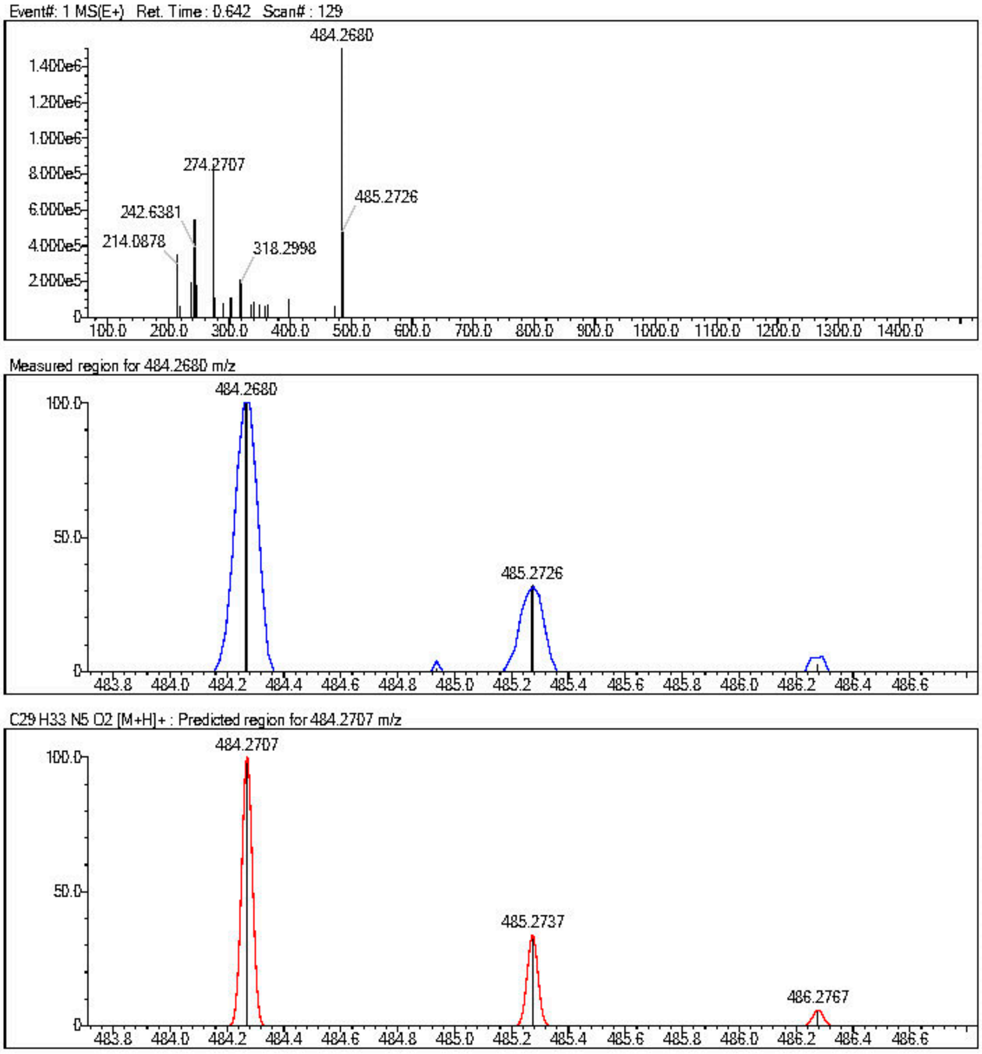

Rank	Score	Formula (M)	Ion	Meas. m/z	Pred. m/z	Df. (mDa)	Df. (ppm)	Iso	DBE
1	84.20	C29 H33 N5 O2	[M+H]+	484.2680	484.2707	-2.7	-5.58	100.00	16.0

HRMS spectrum of **QUCO-1**

<Chromatogram>

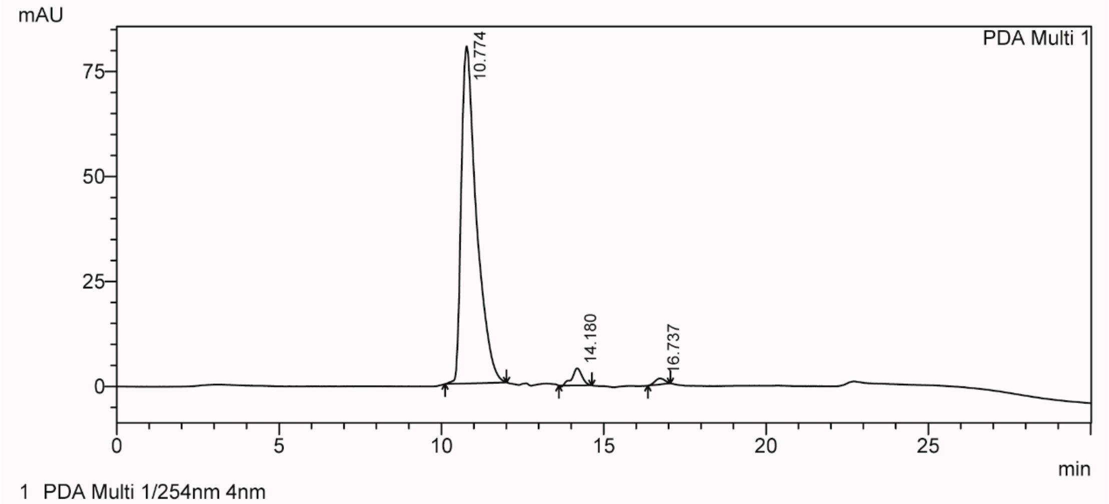

PeakTable

PDA Ch1 254nm 4nm

Peak#	Ret. Time	Area	Height	Area %	Height %
1	10.774	2614113	80303	95.673	93.577
2	14.180	88086	4094	3.224	4.771
3	16.737	30153	1418	1.104	1.652
Total		2732352	85815	100.000	100.000

HPLC spectrum of **QUCO-1**
